# Challenges in the Pediatric Celiac Disease Diagnosis: An Up-to-Date Review

**DOI:** 10.3390/diagnostics15182392

**Published:** 2025-09-19

**Authors:** Alexandra Mpakosi, Christiana Kaliouli-Antonopoulou, Vasileios Cholevas, Stamatios Cholevas, Ioannis Tzouvelekis, Maria Mironidou-Tzouveleki, Alexandra Lianou, Nicoletta Iacovidou, Andreas G. Tsantes, Rozeta Sokou

**Affiliations:** 1 Department of Microbiology, General Hospital of Nikaia “Agios Panteleimon”, 18454 Piraeus, Greece; 2 Department of Immunology, General Hospital of Nikaia “Agios Panteleimon”, 18454 Piraeus, Greece; ckalanto@gmail.com; 3 School of Medicine, University of Bologna, 40126 Bologna, Italy; billcholevas34@gmail.com; 4 School of Pharmacy, European University of Cyprus, Diogenes, Engomi, 2404 Nicosia, Cyprus; stam17112004@gmail.com; 5 School of Agricultural Technology, Food Technology and Nutrition, Alexander Technological Educational Institute of Thessaloniki, 57400 Thessaloniki, Greece; tzouvelekisgiannis@yahoo.gr; 6 Department of Pharmacology, School of Medicine, Faculty of Health Sciences, Aristotle University of Thessaloniki, 54124 Thessaloniki, Greece; mmyronidauth@gmail.com; 7 Neonatal Intensive Care Unit, General Hospital of Nikaia “Agios Panteleimon”, 18454 Piraeus, Greece; alexlianou95@gmail.com; 8 Neonatal Department, Aretaieio Hospital, National and Kapodistrian University of Athens, 11528 Athens, Greece; niciac58@gmail.com; 9 Laboratory of Haematology and Blood Bank Unit, School of Medicine, “Attiko” Hospital, National and Kapodistrian University of Athens, 12462 Athens, Greece; andreas.tsantes@yahoo.com; 10 Microbiology Department, “Saint Savvas” Oncology Hospital, 11522 Athens, Greece

**Keywords:** celiac disease, transglutaminase, endomysial antibodies, gluten-free diet

## Abstract

Celiac disease (CD) is an autoimmune disorder that affects genetically susceptible individuals, characterized by specific serological and histological features, and is triggered by the consumption of gluten. The current diagnosis is based on the demonstration of intestinal damage in small bowel biopsies, as well as the serological presence of CD-specific antibodies (usually IgA) against tissue transglutaminase (tTG), deamidated gliadin peptides (DGP), and endomysium (EMA). The European Society for Paediatric Gastroenterology, Hepatology and Nutrition (ESPGHAN), in the 2020 updated guidelines, states that the diagnosis of CD in children and adolescents can be established without a biopsy if they have IgA tTG2 >10 ULN confirmed by positive IgA endomysial antibodies on two separate blood tests. Challenges, though, arise in serological and clinical diagnosis: in several cases false-positive results are observed. False-negative serological tests may also occur in children < 2 years of age, in patients adhering to a gluten-free diet, in individuals on immunosuppressive therapy, in cases of selective IgA deficiency, and finally due to potential laboratory errors. CD has a wide range of clinical manifestations, either gastrointestinal or extraintestinal. However, CD may be clinically silent and diagnosed through screening. Delayed diagnosis and treatment can lead to serious complications. Therefore, understanding and awareness of these challenges is imperative. Hence, the aim of this review is to highlight the diagnostic challenges of celiac disease in children and adolescents and stress the importance of prompt recognition in order to ensure appropriate management and prevention of complications.

## 1. Introduction

Celiac disease (CD) is a chronic immune-mediated enteropathy that affects genetically susceptible individuals [human leukocyte antigen (HLA)-DQ2 and/or HLA-DQ8] in children and adults. It is one of the most common disorders in Western countries and is associated with gluten intake (found mainly in wheat, rye, and barley). The diagnosis is based on clinical manifestations, the presence of specific antibodies, and enteropathy confirmed by biopsy [1]. Early diagnosis is very important, not only because it improves patients’ clinical symptoms, but also because it reduces the likelihood of serious complications [2].

CD has a wide range of clinical manifestations, gastrointestinal and extraintestinal. The classic manifestations of CD occur most frequently in children and very rarely in adults [3,4]. In younger children, the most common gastrointestinal symptoms are diarrhea, abdominal distension, anorexia and abdominal pain. In older children, depending on the amount of gluten intake, gastrointestinal symptoms include diarrhea and/or constipation, nausea, vomiting, abdominal distension and abdominal pain [5]. As for the extraintestinal symptoms, if the disease remains undiagnosed for a long time, there may be growth retardation, weight stagnation, short stature, malnutrition, recurrent oral aphthae, tooth enamel hypoplasia, iron deficiency anemia, and/or other deficiencies (vitamin B12, folic acid, calcium, and vitamin D), osteopenia, osteoporosis, fatigue, irritability, ataxia, epilepsy, anxiety, and depression [6]. However, nowadays the classical manifestations of CD-severe malnutrition are rare, even in infants. In adolescence, symptoms are usually atypical. Hypogonadism in girls and delayed puberty in boys may be the only manifestations of the disease during this period [7]. Unexplained infertility, miscarriages and recurrent abortions are adult manifestations [8]. In addition, up to 20% of CD patients are asymptomatic, especially patients diagnosed through screening of related diseases [9,10].

The serological diagnosis of CD is based on specific antibodies that include tTG IgA and IgG antibodies, EMA IgA and IgG antibodies, and deamidated gliadin peptide IgA and IgG antibodies. There are also classic gliadin antibodies, the use of which is now outdated and is not recommended by ESPGHAN [11]. tTG IgA antibodies are the serological markers of choice [1]. Given their high sensitivity and specificity, ESPGHAN recommends that diagnosis of CD in children and adolescents can be made without a biopsy, whether or not the child has symptoms, provided that tTG IgA levels are at least ten times the upper limit of normal, and EMA antibodies are positive on two separate blood tests [11]. However, in cases of IgA deficiency, the presence of tTG IgG or EMA IgG antibodies should be determined [11]. Antibodies against deamidated gliadin peptide (DGP) are less sensitive and specific than antibodies against tTG. In all IgA-deficient patients the diagnosis of CD must be confirmed by histological examination. Several intestinal biopsy samples (two from the bulb and four from the distal duodenum) must be obtained to confirm the final result [12].

Due to the wide range of symptoms, CD diagnosis may be difficult, requiring a high index of suspicion [13]. In rare cases with negative serological tests or in doubtful cases other methods that can be used include determinations of intraepithelial lymphocytes with T-Cell Receptor (TCR γδ+), subepithelial deposits of tTG IgA in the intestinal mucosa and blood vessels, interferon (IFN)-c-secreting T cells reactive to gluten (in the peripheral blood of patients after short-term consumption of gluten), serum IL-2, IL-8, and IL-10 (increased levels in CD), and specific lymphocytes in peripheral blood [14,15,16,17].

The diagnosis of pediatric CD is often difficult due to the presence of non-specific manifestations. Whitburn J et al. reported that estimates of undiagnosed cases in socioeconomically deprived children may be at least 91%, while for children with a socioeconomic advantage, the rate may be at least 83%. The authors suggested reconsideration of mass serological testing of children commencing primary school [18]. In resource-limited countries, confirmatory diagnosis is difficult due to the poor availability of standardized anti-tTG tests, and to the difficulties in performing pediatric biopsies, as a result of lack of appropriate facilities and lack of experience [19]. This up-to-date review attempts to highlight the challenges in the pediatric CD diagnosis and to sensitize pediatricians and primary care providers to be vigilant in order to promptly identify suspicious clinical cases so that patients receive appropriate treatment, their symptoms are alleviated, and complications are avoided.

## 2. Epidemiology of Pediatric CD

In recent decades, there has been a global increase in the prevalence of pediatric CD, with rates reaching up to 1.65% [20]. This increase can be attributed partly to the availability of reliable serological tests, greater awareness, and various environmental predisposing factors that increase the risk of developing the disease in genetically susceptible individuals. Variations in the incidence of the disease worldwide are recorded, attributed to different environmental, genetic and epigenetic factors [21]. For example, in countries with low socioeconomic backgrounds, CD is often underdiagnosed mainly due to limited access to diagnostic methods, misinterpretation of serological and histopathological tests, low awareness and alertness of clinicians regarding the symptoms, patient unawareness, and the lack of standardized diagnostic and treatment protocols [22].

## 3. Environmental Factors Involved in the Risk of CD in Children

The disease affects only individuals who carry certain risk haplotypes of HLA, such as the HLA-DQ2 or DQ8. However, although approximately 40% of the general population carry these genes, only around 3% actually develop the disease. Even among monozygotic twins, the concordance reaches only 80% [23]. Therefore, it appears that in addition to gluten, other environmental factors are implicated in the pathogenesis of the disease. The mode and timing of delivery, neonatal and childhood infections, infant feeding practices, consumption of other wheat proteins, and antibiotic use, are implicated in influencing the risk of developing CD in children. The use of certain medications and a gluten-containing diet by the mother during pregnancy are likely to contribute to the risk of the disease in the offspring. In contrast, a mother’s diet rich in fiber is likely to be associated with a low risk [24,25]. Some studies also indicate delivery by that cesarean section is particularly implicated in the risk of the disease possibly, due to reduced colonization of the newborn gut by maternal vaginal microbiota, which can adversely affect the development of the intestinal microbiome [26,27]. However, other studies reported no association between cesarean delivery and CD [28,29].

Seasonal environmental exposures may also play an important role in the risk of developing CD. It has been hypothesized that CD is more likely to develop in children born in spring or summer, due to greater viral exposure during pregnancy or because these infants are typically weaned and introduced to gluten during autumn and winter, a period associated with higher exposure to seasonal viral infections [30]. On the contrary, other studies showed that autumn births are associated with a higher risk of developing the disease, unlike summer births [31].

The duration of breastfeeding has not been clearly linked to the risk of developing the CD, and no significant difference was observed between children who were breastfed and those who were not [32]. However, breastfeeding during the initial introduction of gluten likely helps reduce the risk of disease development.

Earlier gluten introduction in the diet of a baby, may be associated with an earlier onset of CD, and higher gluten intake during or after weaning could increase disease risk [33]. In contrast, other studies report that introducing gluten earlier, at around 4 months rather than the typical 6 months, may be linked to a lower risk of developing CD [34]. Data from the TEDDY study reported considerable variation in the timing of gluten introduction across countries, as well as differences in the risk of developing CD autoimmunity and CD [35]. Nevertheless, the age at first gluten introduction does not appear to independently predict CD by 5 years of age, either overall or in country-specific comparisons.

Infections may also be implicated in the risk of CD through different molecular mechanisms [36,37]. Rotavirus, which is the leading cause of acute gastroenteritis in infants and children < 5 years of age, is implicated in the pathogenesis of CD, likely through “molecular mimicry” of its human capsid VP7 antigen, which is recognized by a subgroup of IgA transglutaminase [38]. Other viruses that were implicated as potential triggers for CD, are adenovirus, enterovirus and reovirus [39,40]. In general, gastrointestinal infections in childhood can cause damage to the mucosal barrier, resulting in gluten transmission [41]. Similarly, microbiome dysregulation and gut dysbiosis can cause barrier disruption, dendritic cell activation and upregulation of tTG in the inflamed small intestine [42]. However, some infections may contribute to the suppression of autoimmunity through mechanisms that include modification of gluten epitopes, shifting the immune response from Th1 to Th2, or reducing immune responses [43].

Exposure to antibiotics during pregnancy and in early childhood, especially before two years of age, may cause long-term alterations in the infant gut microbiome and potentially increase the risk of CD development [44]. Proton pump inhibitors (PPIs), medications that suppress gastric acid secretion and alter gastric pH, are often used in pediatric patients, and have also been implicated in increasing CD risk [45].

Vitamin D deficiency is implicated in the risk of developing CD. Vitamin D protects the intestinal mucosa from damage caused by gluten intake by reducing the Th1 response. In addition, it contributes to the maintenance of the integrity of epithelial permeability by increasing the expression of proteins responsible for tight junctions [46]. High levels of vitamin D in children may cause intense immune activation and increased activity of inflammatory mediators and toll-like receptors, also increasing the risk of CD [47,48].

## 4. Immunological Mechanisms of CD

**Figure 1:**
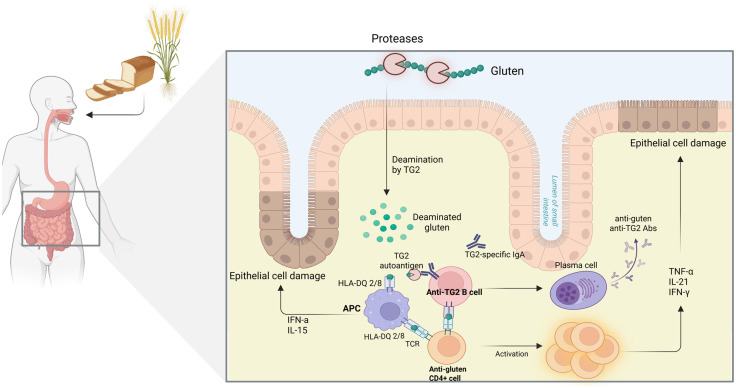
**Immunological mechanisms of CD.** APC: Antigen-presenting cell, HLA: Human leukocyte antigen, IL: Interleukin, IFN: Interferon, TNF: Tumor necrosis factor, TG2: Transglutaminase 2, TCR: T-cell receptor. Created in BioRender. L., A. (2025) https://BioRender.com/zy3znxq, accessed on 20 August 2025. Clinical manifestations of pediatric CD.

Gluten peptides enter the lamina propria and are deamidated by transglutaminase 2 (TG2). The deamidated gluten peptides then bind to specific HLA-DQ2/HLA-DQ8-expressing antigen-presenting cells and are recognized by CD4+ T-cells. The subsequent overactivation of inflammatory cytokines in the mucosa of patients with CD, such as interleukin (IL)-15 or interferon (IFN)-α, leads dendritic cells to acquire an inflammatory phenotype and secrete IL-12, triggering the differentiation of TH1 CD4+ T cells that produce IFN-γ and IL-21. IFN-γ contributes to the function of CD8+ T cells and natural killer (NK) cells, while IL-21 promotes the humoral response and Th17-type immunity [49]. Therefore, T and B cells interact with each other, leading to the differentiation of plasma cells and the production of antibodies against TG2 and against deamidated gluten peptides, as well as the accumulation of effector CD4+ T cells in the lamina propria, which play a key role in the activation of cytotoxic intraepithelial lymphocytes and tissue destruction [50]. IL-17A and tumor necrosis factor (TNF), promote the secretion of matrix metalloproteinases (MMPs) from stromal cells, resulting in extracellular matrix (ECM) degradation and epithelial damage (Figure 1) [49].

## 5. Clinical Presentations of CD

CD presents with a wide spectrum of symptoms, which can vary greatly between patients. Manifestations may arise from the gastrointestinal tract, including diarrhea, abdominal pain, and malabsorption, or may be extraintestinal, such as anemia, growth failure, neurological symptoms, or dermatological signs. In many cases, patients may exhibit a combination of both intestinal and extraintestinal features, while some remain asymptomatic despite characteristic intestinal damage. Gastrointestinal symptoms are more common in younger children, in contrast to extraintestinal manifestations, which occur more frequently in older ones. Similarly, levels of autoantibodies against CD-specific tissue transglutaminase at the time of diagnosis may be higher than 10 times the upper limit of normal in younger children than in older ones [51]. CD is diagnosed more frequently in females, with a reported female-to-male ratio ranging from 2:1 to 3:1 [52,53]. However, serological screening suggests a true ratio closer to 1.5:1 [54]. The disease has no specific age of onset, and it may range from early childhood to late adulthood. Nevertheless, the onset of CD is most frequently either within the first two years of life—the period of weaning and gluten introduction—or in the second or third decade of life. Diagnosis can be difficult due to the great variability of clinical manifestations among patients [55]. The disease is considered a “Chameleon”; many patients are asymptomatic, without any manifestation of malabsorption. We still cannot explain the variability in the presentations of CD.

### 5.1. Gastrointestinal Manifestations

As mentioned before, abdominal pain, bloating, and chronic or intermittent diarrhea are among the main symptoms of the gastrointestinal system. Loose stools may be bulky, greasy, and have an unpleasant odor. However, long-term constipation due to impaired motility of the upper gastrointestinal tract can also be the main manifestation in both children and adults [56,57]. Infants and young children may also experience vomiting, decreased appetite, weight loss, and difficulty growing, symptoms that can even lead to malnutrition and wasting [58]. CD may also occur in overweight/obese children as a result of the overweight/obesity epidemic [59,60].

An association between eosinophilic esophagitis (EoE) and CD was reported [61,62]. The risk of developing CD in children with EoE is reported high in published data, with rates ranging from 9% to 35% [63,64]. EoE may be a finding on an upper endoscopy for CD.

In addition, very young children with undiagnosed CD may rarely develop intussusception. Even more rarely, an acute episode of diarrhea and enteropathy with protein loss, hypoalbuminemia, severe electrolyte disturbances, metabolic acidosis and hypokalemia accompanied by dehydration and lethargy, a condition known as “celiac crisis”, is reported in young children [65].

### 5.2. Extraintestinal Manifestations

CD is often underdiagnosed, in part due to the wide range of its extraintestinal manifestations that can mislead and delay diagnosis.

#### 5.2.1. Oral Manifestations

Recurrent aphthous stomatitis, delayed dental eruption and maturation, dental enamel defects, and geographic tongue may be observed. Dental enamel hypoplasia is observed in up to 40–50% of newly diagnosed pediatric celiac patients, compared with about 6% in healthy children [66]. In some cases, these may be the only manifestations of the disease. Several factors are involved, including immunological, genetic, atopic, nutritional, endocrine, stress, and local trauma (especially in recurrent aphthous stomatitis) [67,68]. Other oral manifestations also include atrophic glossitis, glossodynia, angular cheilitis, and salivary gland abnormalities.

#### 5.2.2. Growth Impairment

Failure to thrive and short stature are frequently reported as extraintestinal manifestations in pediatric CD [69,70,71]. Malnutrition due to malabsorption is implicated as the main cause of growth retardation, while dysfunction of the growth hormone (GH)-insulin-like growth factor (IGF1) axis plays a role [71]. Starting a strict gluten-free diet before puberty lead to catch-up growth within 2 years. However, this is difficult to achieve after puberty, i.e., in the case of late diagnosis of the disease. The lack of response to a strict gluten-free diet could mask another comorbidity, including inflammatory bowel disease, Turner syndrome, or growth hormone deficiency [58].

#### 5.2.3. Elevated Liver Enzymes Which Decline on Gluten-Free Diet Without Being Autoimmune Hepatitis

Hypertransaminemia is associated with increased passage of hepatotoxins into the portal circulation due to leaky gut, resulting in inflammation and liver damage, as well as deposition of anti-tTG antibodies in the liver [71,72,73]. Usually, this liver damage is reversible after a gluten-free diet program, although rare cases that result in liver failure cannot be ruled out [74].

#### 5.2.4. Delayed Puberty

It is a relatively common manifestation in pediatric CD, affecting approximately 10% of newly diagnosed patients. In girls, this includes failure to develop breasts by age 13 or, if development occurs, absence of menstruation after three years or by age 16. In boys, its main characteristics are the lack of testicular enlargement by the age of 14, or a delay of five years or more after the onset of genital development. In CD, such delays are primarily attributed to malabsorption and nutritional deficiencies [75]. A strict gluten-free diet can restore normal maturation within usually 12–24 months [58].

#### 5.2.5. Anemia

(A) Iron-deficiency anemia is among the most frequent extraintestinal manifestations in adults with CD, present approximately in 50% at diagnosis, whereas it occurs in only about 10–15% of newly diagnosed pediatric patients [75]. Iron deficiency anemia is mainly due to atrophy of the mucosal villi in the duodenum, where iron is absorbed, leading to inflammation and activation of the mechanisms of anemia of chronic disease [76]. It may be the only clinical manifestation of the disease, especially in older children. More often, it is accompanied by increased serological markers and indicates severe histological damage [77]. A gluten-free diet and/or iron supplements, if needed, usually correct the condition. However, some patients continue to have iron deficiency anemia despite proper adherence to gluten-free diet and normalization of villous atrophy. Other patients are even resistant to oral iron supplementation despite mucosal healing and, therefore, require intravenous iron administration [76]. In children, intravenous iron therapy can rapidly replenish iron stores, resolution of anemia, and reduce gastrointestinal side effects [78].

(B) Other types of anemia can also be caused by folate or vitamin B12 deficiencies, by blood loss, and through co-occurrence with inflammatory bowel disease (IBD) or other diseases. Vitamin B12 deficiency may also be responsible for the development of peripheral myeloneuropathy. Folate deficiency is more common in adults than in children [79].

#### 5.2.6. Osteopenia

It is characterized by reduced bone density, and osteoporosis, which results in fragile and frail bones and are the most common bone-related complications of CD. If left untreated, these conditions lead to increased bone brittleness and higher risk of fractures. At diagnosis, approximately 75% of pediatric celiac patients exhibit osteopenia, while around 30% present with osteoporosis [80].

Vitamin D deficiency or insufficiency (serum 25-hydroxyvitamin D levels less than 20 ng/mL or 20–30 ng/mL, respectively) is the most common vitamin deficiency in children with CD. It is mainly due to intestinal malabsorption and chronic inflammation and is associated with low bone density [81]. There is no apparent correlation between such deficiencies and serological tests or mucosal damage. Children with CD achieve restoration of most serum micronutrient levels after 6 months on gluten-free diet, in contrast to vitamin D, whose levels may improve but remain below normal limits [82]. Low bone density may be restored within 1 year of starting a gluten-free diet [81]. According to current recommendations from ESPGHAN, regular bone mineral density screening is not recommended in children with CD, because reduced bone mineral density is not associated with an increased risk of fracture [83].

#### 5.2.7. Musculoskeletal Manifestations

Arthralgia and arthritis, occur in approximately 5–10% of newly diagnosed pediatric celiac patients [84]. The knee is the most commonly affected joint, and finding include joint effusion, synovial hypertrophy, and synovitis [85,86]. Adhering to a gluten-free diet for at least six months can lead to improvement of these articular manifestations [85,86].

#### 5.2.8. Neurological Manifestations

Neurological manifestations are also reported. Various mechanisms related to gluten and subsequent pathogenesis are involved, such as antibody cross-reactivity, immune complex deposition, direct neurotoxicity, vitamin or nutrient deficiency, and the gut microbiome through the “gut-liver-brain axis” [87]. The most frequent neurologic manifestations are headache, migraine, peripheral neuropathy, epileptic seizures, intellectual disability, ataxia and attention deficit and hyperactive disorder [88]. Among them, headache is usually reported in children with CD in approximately 20–30% of newly diagnosed cases, with higher rates observed in adolescents [85,88]. Adherence to a gluten-free diet may lead to improvement of the symptom within 6–12 months [89,90]. Epilepsy is also reported [91,92]. Generalized tonic–clonic seizures are common. A few cases of epilepsy associated with symmetrical occipital calcifications, mainly from Italy, Spain, and Argentina, were reported [93]. This type of epilepsy can rarely progress to epileptic encephalopathy [93]. However, a gluten-free diet can improve seizures, even in cases of epilepsy resistant to antiepileptic drugs [87].

#### 5.2.9. Psychiatric Manifestations

**Figure 2:**
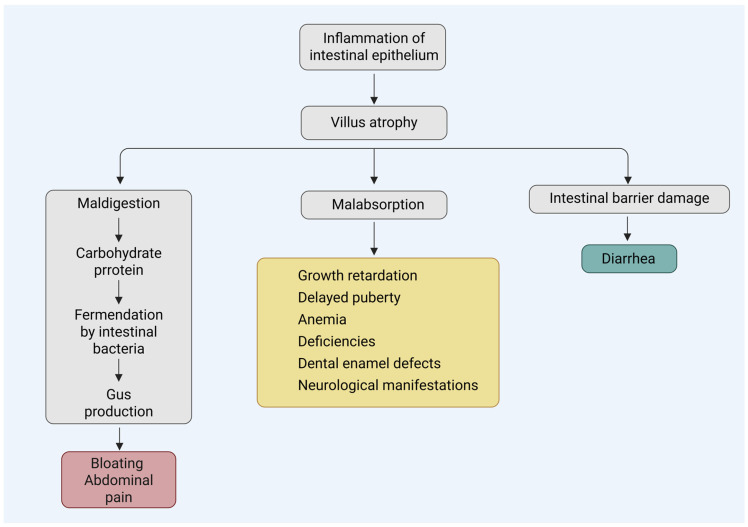
Mechanisms of the main clinical manifestations of pediatric CD. Created in BioRender. L., A. (2025) https://BioRender.com/kxm4o3q, accessed on 20 August 2025.

Anxiety, depression, panic attacks, hallucinations, cognitive and behavioral issues, are also frequently reported in children and especially adolescents with CD [94,95]. Introducing a gluten-free diet can also lead to improvement of such symptoms (Figure 2) [96].

#### 5.2.10. Dermatitis Herpetiformis

The skin is frequently affected in CD and the main manifestation is dermatitis herpetiformis, characterized by papules and urticarial plaques mainly on the elbows, knees, and buttocks [97]. However, children with dermatitis herpetiformis may have atypical skin manifestations, such as isolated hemorrhagic lesions on the palms, soles, and face, as well as deep cutaneous nodules [98,99]. The diagnosis of dermatitis herpetiformis is made by direct immunofluorescence (IF), which shows granular IgA deposits in the papillary dermis and the dermo-epidermal junction. The tTG3 isoenzyme found in the skin (epidermal transglutaminase) is the target of the autoantibodies. A gluten-free diet results in the repair of skin damage [100]. Although rare, this is a serious condition that should be included in the pediatrician’s differential diagnosis in children of any age with an atypical rash [101].

## 6. High-Risk Groups

### 6.1. First-Degree Relatives

The risk of developing the disease in first-degree relatives of celiac patients can reach 10–20% [102]. The prevalence of the disease in monozygotic twins can reach up to 75–80% [103].

### 6.2. Patients with Associated Disorders

#### 6.2.1. Associated Autoimmune Diseases

A significant group of genes that CD and other autoimmune diseases have in common, belong to the HLA family [104]. Thus, there is an association between CD, type 1 diabetes (T1D), thyroid disease, gastric autoimmunity leading to pernicious anemia, vitiligo, and adrenal insufficiency [105]. Prolonged exposure to gluten may potentially play a role in the development of other autoimmune disorders [58,106].

Usually, the onset of T1D precedes CD. Most children have elevated tTG-IgA levels at the time of diagnosis of T1D, but may be diagnosed with CD several years after the onset of T1D [107]. Furthermore, patients with T1DM may have persistent low to moderate positive tTG-IgA titers regardless of the presence of CD. A confirmatory biopsy is recommended if these titers are more than three times the upper normal limit. If there is no mucosal damage on biopsy, these cases are characterized as “false positive” tTG-IgA or as “potential CD” [58,108]. On the other hand, CD is usually asymptomatic in children with diabetes. Therefore, it is recommended that children belonging to such risk groups undergo repeated screening. A gluten-free diet improves elevated HbA1c levels, contributes to better glycemic control, and reduces the incidence of micro- and macrovascular complications, and the episodes of ketoacidosis and hypoglycemia [109].

Hashimoto’s thyroiditis more commonly, and Graves’ disease, are autoimmune thyroid diseases associated with CD. The presence of antithyroid antibodies in children with CD is possible, but they probably have low predictive value for the development of autoimmune thyroiditis in later years [110]. Symptoms, such as fatigue, weight loss, constipation or diarrhea, and arthritis seropositive or not, may also be common in both autoimmune thyroiditis and CD [111]. Therefore, vigilance and screening for CD are required in all children and adolescents with autoimmune thyroiditis and vice versa [112].

Autoimmune hepatitis was also reported in children with CD. Introduction of a gluten-free diet is likely to improve symptoms [113].

An association between idiopathic juvenile arthritis and CD was reported. Therefore, screening for CD is recommended in all children with juvenile idiopathic arthritis [114].

Children with CD are at increased risk for autoimmune skin disorders as well, such as psoriasis, vitiligo, and alopecia areata. It is unclear whether a gluten-free diet has any positive effects on these skin disorders, except perhaps psoriasis, whose symptoms may improve [115].

#### 6.2.2. Associated Genetic Diseases

Patients with Down syndrome, Turner syndrome, and William–Beuren syndrome are at high risk of developing CD and should therefore be screened [116,117,118].

## 7. Serological Testing

### 7.1. Serological Diagnosis of Pediatric CD

In CD, gluten deamidation is caused by the intervention of small intestinal transglutaminase against which B-cells can produce antibodies [119]. Therefore, serological testing for CD is the first test performed when the disease is suspected, in symptomatic or asymptomatic children of high-risk groups. At the same time, and while still on gluten-containing diet, total IgA should also be measured [11,120,121,122]. The revised 2020 ESPGHAN guidelines recommend that: (a) tTG IgA is as a more reliable marker of the disease vs. DGP IgG (which was previously recommended) in individuals with normal serum IgA values, even in young children under 2 years of age [11], and (b) diagnosis of the disease can be safely made for all children, both symptomatic and asymptomatic, even without a duodenal biopsy, under the conditions that tTG IgA levels are more than 10 times higher than the upper limit of the normal range, and EMA IgA antibodies are detected. In contrast to the old 2012 guidelines, the criterion for HLA typing was omitted. The diagnosis can be confirmed by the resolution of symptoms and normalization of the IgA tTG titer, when a gluten-free diet is introduced.

Although the tTG test has high sensitivity and reproducibility, false-positive results may be observed, usually occurring at low titer values and up to about twice the diagnostic limit. Such results should be evaluated in relation to the clinical status of the patient, who should be referred to a CD reference center for long-term follow-up [123].

The EMA test has high specificity and is often used as a confirmatory test for the disease in individuals with a positive tTG test [124]. However, the detection of EMA antibodies by indirect immunofluorescence depends on the experience of the observer and has differences in the standards followed by various laboratories in the interpretation of the EMA test results [125,126].

Nevertheless, the diagnosis of CD based on serology alone, should be made by a pediatric gastroenterologist, and only after discussing with the family advantages and disadvantages of each method: the disadvantages of diagnosis without biopsy may include false-positive tests, potential CD, and questionable long-term compliance with gluten-free diet. Advantages of endoscopy include diagnostic accuracy, and the option of diagnosing concurrent diseases (such as EoE).

**Figure 3:**
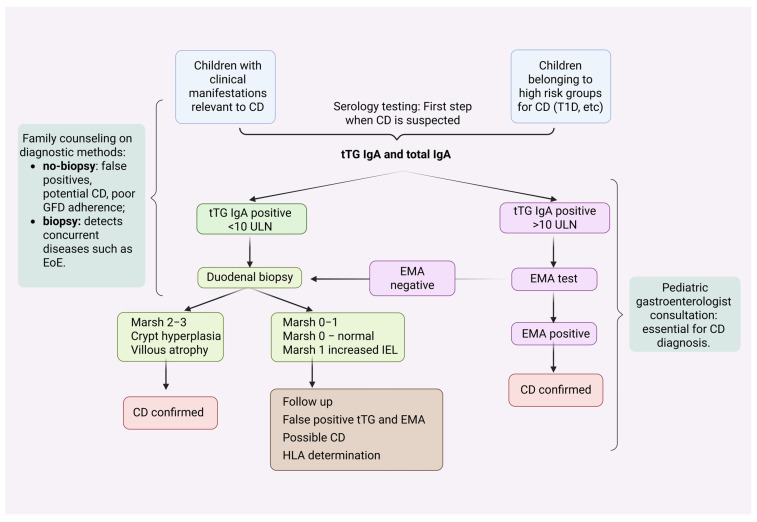
Algorithm for the diagnosis of CD in case of a positive serological test. CD: Celiac Disease, tTG IgA: Tissue transglutaminase IgA antibodies, EMA: Endomysial antibodies, T1D: Type 1 diabetes, IEL: Intraepithelial lymphocytes, HLA: Human leukocyte antigen; GFD: gluten free diet, EoE: Eosinophilic Esophagitis. Created in BioRender. L., A. (2025) https://BioRender.com/fyg15lb, accessed on 20 August 2025.

If histological confirmation is required (for example, in the case of seronegative CD or if tTG-IgA is positive but with titers <10 times the upper limit of normal), the patient must have consumed a sufficient amount of gluten at the time of biopsy, and a sufficient number of duodenal biopsy specimens must be obtained. If the result of the histological examination is normal, despite the positive serological test, the case is considered potential CD (Figure 3) [127].

Nevertheless, it should be taken into account that villous atrophy on biopsy can also be observed in various other enteropathies, such as autoimmune enteropathy, Giardia lamblia infection, Crohn’s disease, tropical inflammation, gastroenteritis, Whipple’s disease, intestinal lymphoma, and human immunodeficiency virus (HIV) enteropathy [128,129,130,131].

### 7.2. Seronegative CD

Seronegative CD is very rare in children. It is characterized by negativity of tissue transglutaminase antibodies, villous atrophy on duodenal biopsy and HLA-DQ2/DQ8 haplotype test [132]. Seronegativity is attributed either to deposits of tTG/anti-tTG immune complexes in mucosal tissues, which do not allow the passage of anti-tTG into the bloodstream, or to incomplete maturation of plasma cells and therefore to the lack of antibody production [132]. The diagnosis of seronegative CD can be confirmed after improvement of both clinical symptoms and histological lesions after 1 year on a gluten-free diet [133].

False-negative serological tests for CD may occur in children under two years of age, in individuals following a gluten-free diet or using immunosuppressants, in children with IgA deficiency, and due to laboratory errors [126,134]. Children who have tested negative for anti-tTG should be monitored for a long period of time due to the possible risk of developing CD later in life. Children belonging to special risk groups, such as type 1 diabetics, should be screened annually [135,136]. Late seroconversion of negative individuals, especially those with risk factors, was observed in long-term follow-up studies [137]. However, if tTG IgA is negative and there is no IgA deficiency, the risk for CD is very low. In rare cases, seronegativity may be due to short-term gluten consumption, low dose or amount of gluten exposure, intermittent gluten intake, or extraintestinal manifestations [138,139,140]. It is worth noting at this point, that in recent years, gluten-free diet has become very popular and is promoted by the media as a healthier way of eating, is followed by people without CD or other related disorders, and has become particularly popular among famous athletes [141,142,143].

### 7.3. Selective IgA Deficiency

Immunoglobulin A (IgA) represents more than 70% of total immunoglobulins. It is found as dimers in mucosal barriers, mainly in the gastrointestinal and respiratory systems, where it plays an important role in the defense against infections, and as monomers in serum at levels that vary with age [144,145].

Total serum IgA levels may be affected by factors such as stress, depression, diet, and lifestyle [146,147,148,149,150]. Male gender, older age, non-Caucasian ethnicity, alcohol consumption, hypertension, and obesity are associated with increased total serum IgA [151]. Elevated levels are found in various diseases such as IgA nephropathy, Henoch-Schoenlein purpura, alcoholic cirrhosis, chronic spinal cord injury, multiple myeloma, monoclonal gammopathies of undetermined significance [152,153,154,155,156,157]. In addition, multiple sclerosis, IgA blistering diseases, and linear bullous IgA disease are also associated with IgA autoantibodies, IgA immune complexes or elevated IgA [158,159,160]. Excessive production of immunoglobulin A against microbiome is observed in inflammatory bowel disease (IBD) and is associated with the pathology of the disease. Several proinflammatory processes are involved [160]. IL-10 and IL-4 may also induce IgA production in patients with IgA deficiency [161,162]. In one study, patients negative for tTG-IgA antibodies, but with gastrointestinal symptoms compatible with CD, were found to have a higher incidence of elevated total serum IgA compared with healthy controls. The authors hypothesized that this was due to either a diet that alleviated the patients’ symptoms, or the stress they experienced due to their clinical condition [157].

**Figure 4:**
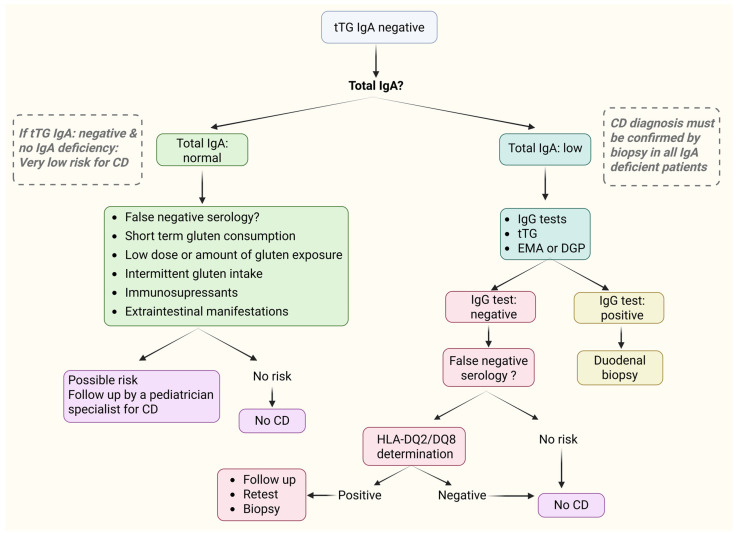
Algorithm for the diagnosis of CD in case of a negative serological test. CD: Celiac disease, tTG IgA or IgG: Tissue transglutaminase IgA or IgG antibodies, EMA: Endomysial antibodies, HLA: Human leukocyte antigen, DGP: Deamidated gliadin peptides. Created in BioRender. L., A. (2025) https://BioRender.com/yazw95b, accessed on 20 August 2025.

Children after birth and up to the age of 4 years, may experience transient developmental IgA deficiency, due to delayed maturation of the IgA system [163]. Beyond 4 years of age, selective IgA deficiency (SIgAD) is defined when serum IgA levels are <0.07 g/L, with normal IgG and IgM levels, and provided that other causes of hypogammaglobulinemia are excluded [119,163]. When total serum IgA levels are low or absent, IgG antibodies such as anti-tTG IgG (or anti-DGP IgG) are evaluated in the diagnosis of CD (Figure 4) [11,164]. Current ESPGHAN guidelines in such cases, state that tTG IgG has great diagnostic value due to its high sensitivity and specificity (84–97% and 91–93%, respectively) [11,120,165]. However, elevated IgG tTG levels can also be detected in other autoimmune disorders such as antiphospholipid syndrome, giant cell arteritis, and ulcerative colitis [166]. Therefore, all cases of IgA deficiency require a biopsy. In addition, IgG is mainly associated with immunological memory, it is more systemic than IgA which is mainly found in mucosal tissues, and therefore recovers more slowly than that [167]. In patients with IgA deficiency and CD, the decrease in IgG tTG is very slow and most of them may still be positive after more than 2 or 3 years on a gluten-free diet [168].

### 7.4. Symptom Resolution and Normalization of Serological Titers

ESPGHAN recommends follow-up visits every 6 months until tTG-IgA levels normalize, and then every 12–24 months. During this period, strict adherence to the gluten-free diet, resolution of symptoms, and recovery of patients are monitored [83]. Clinical manifestations tend to improve more rapidly in children than in adults. Gastrointestinal symptoms tend to resolve more rapidly than extraintestinal symptoms. However, both gastrointestinal and extraintestinal symptoms may persist for a long time even after patients adhere to a gluten-free diet, and for this reason long-term follow-up is required [169,170]. In general, catch-up of developmental delay in preadolescent children should occur within about a year [89].

tTG IgA titers also decline slowly and their normalization depends on adherence to the gluten-free diet and the level of initial titers. They may remain elevated even 18–24 months after full compliance with such a diet [171]. In a recent study by Sbravati F et al., normalization of tTG-IgA of pediatric patients was achieved in 67.3%, 80.7%, 89.8% and 94.9%, after 12, 18, 24 and 36 months (median time 9 months), respectively, from the onset of a gluten-free diet. Additionally, factors such as TGA-IgA values >10 times the upper limit at diagnosis, age 7–12 years, poor dietary compliance, female gender, non-Caucasian ethnicity, and any comorbidities, led to a longer time to normalization [172].

Furthermore, it may take over three years on a gluten-free diet for common laboratory parameters, including iron (Fe), calcium (Ca), transaminases, and leukocyte counts, to return to normal levels [173].

Complete healing of the intestinal mucosa can be observed after 6 months to 2 years or more, on a gluten-free diet [174,175]. Higher rates of mucosal healing are observed in females than in males. Age of onset of the disease also plays a role, as patients who are first diagnosed at an older age have lower recovery rates [174,176]. Adult patients show a lower rate of mucosal healing compared to children, even if they fully comply with a strict gluten-free diet [177]. They may also experience persistent villous atrophy despite normalization of serum tTG or EMA levels [138,178].

## 8. Mass Screening of Asymptomatic Children

Mass testing of asymptomatic children in high-risk groups could perhaps lead to early diagnosis of new cases. Many of these “asymptomatic” children may have subtle manifestations and/or laboratory findings that are corrected by a gluten-free diet [179]. It has also been recently argued that children detected through screening may have systemic inflammation and reduced bone mineral density at the time of diagnosis, which can also be treated with diet [180]. Previously undetected iron deficiency anemia and other micronutrient deficiencies can also be identified in children through mass screening [181]. Furthermore, an increased risk of enteropathy-associated T-cell lymphoma, which belongs to the non-Hodgkin lymphomas, as well as intestinal adenocarcinoma, was reported, mainly in elderly patients with CD, in those who remain undiagnosed for a long time, or in those who have severe malabsorption [182,183,184]. Although these serious complications are rare, early diagnosis through mass screening strategies may potentially reduce this risk.

Several studies from different countries reported the implementation of mass screening programs for CD [185,186], and it was shown that this can be cost-effective, provided that it reduces the risk of future serious complications such as osteoporosis and malignancies, and that individuals identified through screening fully comply with the gluten-free diet [187,188]. In a multicenter Italian study, children aged 5–11 years were screened at school with HLA-DQ2 and DQ8 determination in a drop of blood, and then with total serum IgA and tTG IgA for those who were positive for HLA-DQ2 and/or DQ8. The study revealed that without mass screening, 60% of children with CD would remain undiagnosed [20]. Italy is the only country so far to have approved by law in 2023 the nationwide implementation of screening for CD and T1D in children aged 1–17 [189].

However, such strategies remain controversial. For example, the U.S. Preventive Services Task Force (USPSTF) highlighted the lack of clear evidence regarding the balance of benefits and harms of screening for CD in asymptomatic individuals from the general population and high-risk groups [190]. Furthermore, asymptomatic children who consider themselves “healthy” and are diagnosed through screening, will have to be convinced that they must follow a strict gluten-free diet for life, and this commitment may potentially burden them psychosocially [191].

## 9. Conclusions

Diagnosing CD in children is difficult mainly due to the wide range of symptoms and the interpretation of serological tests. Incorrect or delayed diagnosis, as well as late initiation or failure to adhere to a gluten-free diet, can lead to life-threatening conditions such as cancer. Mass screening of asymptomatic children remains controversial. However, seronegative children exhibiting symptoms suggestive of CD require long-term monitoring due to the potential for later seroconversion. Additionally, children belonging to high-risk groups should undergo annual clinical and serological follow-up.

## Data Availability

Data are contained within the article.
